# Prevalence of *Chlamydia trachomatis, Ureaplasma parvum* and *Mycoplasma genitalium* in Infertile Couples and the Effect on Semen Parameters

**DOI:** 10.4314/ejhs.v33i1.17

**Published:** 2023-01

**Authors:** Khadijeh Ahmadi, Mojtaba Moosavian, Jalal Mardaneh, Omid Pouresmaeil, Maryam Afzali

**Affiliations:** 1 Department of Microbiology, School of Medicine, Abadan University of Medical Sciences, Abadan, Iran; 2 Department of Microbiology, Faculty of Medicine, Ahvaz Jundishapur University of Medical Sciences, Ahvaz, Iran; 3 Department of Microbiology, School of Medicine, Infectious Diseases Research Centre, Gonabad University of Medical Sciences, Gonabad, Iran; 4 Department of Microbiology and Virology, Faculty of Medicine, Mashhad University of Medical Sciences, Mashhad, Iran; 5 Student Research Committee, Faculty of Medicine, Mashhad University of Medical Sciences, Mashhad, Iran; 6 Department of Microbiology, Faculty of Medicine Sciences, Mashhad Medical Sciences, Islamic Azad University, Mashhad, Iran; 7 Department of Laboratory Sciences, Faculty of Paramedicine, Mashhad Medical Sciences, Islamic Azad University, Mashhad, Iran

**Keywords:** Chlamydia trachomatis, Mycoplasma genitalium, Ureaplasma parvum, Infertility, Semen Analysis, Polymerase Chain Reaction

## Abstract

**Background:**

Chlamydia trachomatis, Ureaplasma parvum, and Mycoplasma genitalium are common sexually transmitted microorganisms. Our study aimed to determine the prevalence of C. trachomatis, U. parvum, and M. genitalium in infertile and fertile couples and the effect of these microorganisms on semen parameters.

**Materials and Methods:**

In this case-control study, samples were collected from 50 infertile couples and 50 fertile couples and were subjected to the routine semen analysis and Polymerase chain reaction (PCR).

**Results:**

C. trachomatis and U. parvum were detected in 5 (10%) and 6 (12%) of semen samples from infertile men. Also, out of 50 endocervical swabs from the infertile women, C. trachomatis and M. genitalium were detected in 7(14%) and 4 (8%) of swab specimens, respectively. In the control groups, all of the semen samples and endocervical swabs were negative. Also, in the group of infertile patients infected with C. trachomatis and U. parvum, sperm motility was lower than uninfected infertile men.

**Conclusions:**

The results of this study showed that C. trachomatis, U. parvum, and M. genitalium are widespread among the infertile couples in Khuzestan Province (Southwest of Iran). Also, our results showed that these infections can decrease the quality of semen. For the prevention of the consequences of these infections, we suggest a screening program for infertile couples.

## Introduction

Infertility is a world health problem and about 8 to 12 percent of couples have experienced infertility during their reproductive life. It is defined as the disability to conceive after regular unprotected intercourse for one year. The recent data according to the World Health Organization (WHO) mentioned that, about 48 million couples and 186 million individuals have infertility around the world (1). Many factors can contribute to human infertility, in which sexually transmitted infections (STIs) as one of the major factors. Among bacterial STIs, *Chlamydia trachomatis, Mycoplasma* spp., and *Ureaplasma* spp. playing an important role in both genital infections and infertility (2).

Chlamydial infections are the most prevalent bacterial STIs in the world that cause pathology in both males and females (3). These infections are asymptomatic in 85% – 90% of infected individuals and are associated with urethritis, prostatitis, cervicitis, epididymitis, pelvic inflammatory disease, ectopic pregnancy, and infertility (4, 5). *C. trachomatis* infections are well known to cause female infertility but the role of these infections in the quality of the sperm and male infertility is controversial, however, some studies mentioned *C. trachomatis* can attach and penetrate inside the spermatozoa. So, parameters of sperm such as volume, viability, motility, morphology, and concentration are to be altered (6).

*Mycoplasma* spp. and *Ureaplasma* spp., are belonging to the *Mycoplasma*taceae family. *Mycoplasma*taceae is a family of bacteria that can grow on acellular culture media. Sixteen *Mycoplasma* species were detected in human infections. *U. parvum* and *M. genitalium* are known as urogenital *Mycoplasma* spp. (7). These microorganisms are potentially pathogenic species frequently isolated from the human genitourinary tract. The prevalence of these infections in males and females is high, ranging from 2%-40.5% and 2%-44.3%, respectively (8). In females, *Mycoplasma* spp. can play a role in cervicitis, pelvic inflammatory disease (PID), endometritis, infertility, ectopic pregnancy, bacterial vaginosis, and preterm delivery (9). In males, these infections are associated with non-gonococcal urethritis, prostatitis, epididymitis, and infertility (10).

The effects of these infections on infertility remain unclear but there is some evidence that genital *Mycoplasma* spp. has a negative impact on fertility (11). The relationship between these infections and infertility has been studied in Iran and other countries (10–15). However, some of these studies are restricted to men or women and some do not include the control group, or only one microorganism has been studied. In this study more than one microorganism in infertile couples with the control group compared, and this is also the first study of the prevalence of *U. parvum* and *M. genitalium* in infertile men in southwest of Iran. This study aimed to investigate the prevalence of *C. trachomatis, U. parvum*, and *M. genitalium* in semen and endocervical swabs collected from infertile and fertile men and women and the effect on semen parameters, in Khuzestan Province (southwest of Iran).

## Material and Methods

**Clinical sample collection**: In the case-control study, a total of 50 infertile couples (n=100, 50 men and 50 women) and also, 50 fertile couples (n=100, 50 men and 50 women) as the control group, who were admitted to the Infertility Treatment Center, Ahvaz, Iran, from August 2016 to March 2017 were selected. The number of samples was determined by the statistical consultant according to the prevalence of these microorganisms in the average number of samples used in other similar studies. A total of 50 semen specimens and 50 endocervical swabs from infertile couples, and 50 endocervical swabs, and 50 semen samples from fertile women and men were included in this study. Samples were taken from patients and healthy men aged 20–40 (31+4.1) years. According to this point that, genital *Mycoplasma* spp. are also considered as commensal organisms, the health group also used, to determine the prevalence of genital *Mycoplasma* spp. and compare it with the patients' group (16).

Inclusion criteria for participants were the following: Their infertility was confirmed by an andrologist, and do not use antibiotics for one week before sampling, also for semen samples sexual abstention for at least 2–3 days before the tests. The healthy group who was attending for the checkup and the inclusion criteria were lack of having a history of infertility and first fertility was established in them and they did not have any defects in the sperm parameters and recent antibiotic therapy.

No re-sampling was performed for this study and the remaining laboratory specimens were used. We did not receive personal information from patients' files only informed consent was obtained from each patient and a questionnaire was completed with patient satisfaction. Semen specimens were collected by masturbation into a sterile container. Then, the samples were liquefied at 36 °C for 30 min in an incubator before analysis. Semen analysis was performed according to the outlined by the World Health Organization (6th edition) criteria to determine: pH, motility, volume sperm concentration, normal forms (1). Endocervical samples were collected by using sterile Dacron swabs and were transported to the laboratory in 5 ml PBS (phosphate buffer solution). All of the samples were stored at -70°C until DNA extraction for PCR assay was conducted.

**DNA extraction and PCR method**: The DNA of isolates was extracted using the High Pure PCR Template Preparation kit (Roche Co., Mannheim, Germany). The primer sequences for *C. trachomatis* and genital *M. genitalium* have been listed in Table 1. Each PCR reaction was prepared in 25 µl final volumes containing 10 ρM of each primer (Takapuzist, Tehran, Iran), 1.5 units of Taq DNA polymerase, 1X PCR buffer, 0.2 mM dNTPs, 3mM MgCl2 (Cinagene, Iran), 0.1–0.5 µl DNA template, and water for the remaining volume of 25 µl. DNA amplification was carried out using a thermal cycler (Eppendorf) with a thermal profile as follows: initial denaturation step at 95°C for 5 min, 35 cycles at 95°C for 30 sec, specific annealing temperature mentioned in (table 1) for 30 sec, extension step at 72°C for 30 sec followed by a final extension at 72°C for 10 min.

**Table 1:** Sequence of *C. trachomatis, M. genitalium* and *U. parvum* primers

microorganism	Primer sequences	Length, bp	annealing	Reference
*M. genitalium*	16SrRNA	427	55°C	(38)
	F: 5' TACATGCAAGTCGATCGGAAGTAGC 3'			
	R: 5' AAACTCCAGCCATTGCCTGCTAG 3'			
*U. parvum*	16SrRNA	327	55°C	(39)
	F: 5' AAATCTTAGTGTTCATATTTTTTAC 3'			
	R: 5' GTAAGTGCAGCATTAAATTCAATG 3'			
*C. trachomatis*	*MOMP gene*	180	50°C	(40)
	F: 5' GCCGCTTTGAGTTCTGCTTCC 3'			
	R:5' GTCGAAAACAAAGTCACCATAGTA 3'			

**Electrophoresis**: The PCR products were visualized and photographed under UV light after electrophoresis for 50 min at 100 V through 1.5% agarose gel containing ethidium bromide (1 µg/mL). The genomic DNA from *C. trachomatis* (ATCC VR-885), *M. genitalium* (ATCC 33530), and *U. parvum* (ATCC 27815D) were used as positive control while normal saline was used as a negative control.

**Statistical analysis**: Statistical analysis was performed with the help of SPSS statistical software package V.11.5 and the Chi-Square test. All tests were considered statistically significant when (P < 0.05) was considered statistically significant.

**Ethical consideration**: The study was approved by the Ethics Committee of Ahvaz Jundishapur University of Medical Sciences (AJUMS). Ethics code: IR.AJUMS.REC.1395.161.

## Results

The following results were obtained based on the patients and control group answers to the questionnaires: The mean age of the infertile and fertile men was 33 (25–40) years old and the mean age of the fertile and infertile women was 31 (19–40) years. Significant differences were not found in the age between infertile and control groups (P = 0. 47).

**Figure 1:**
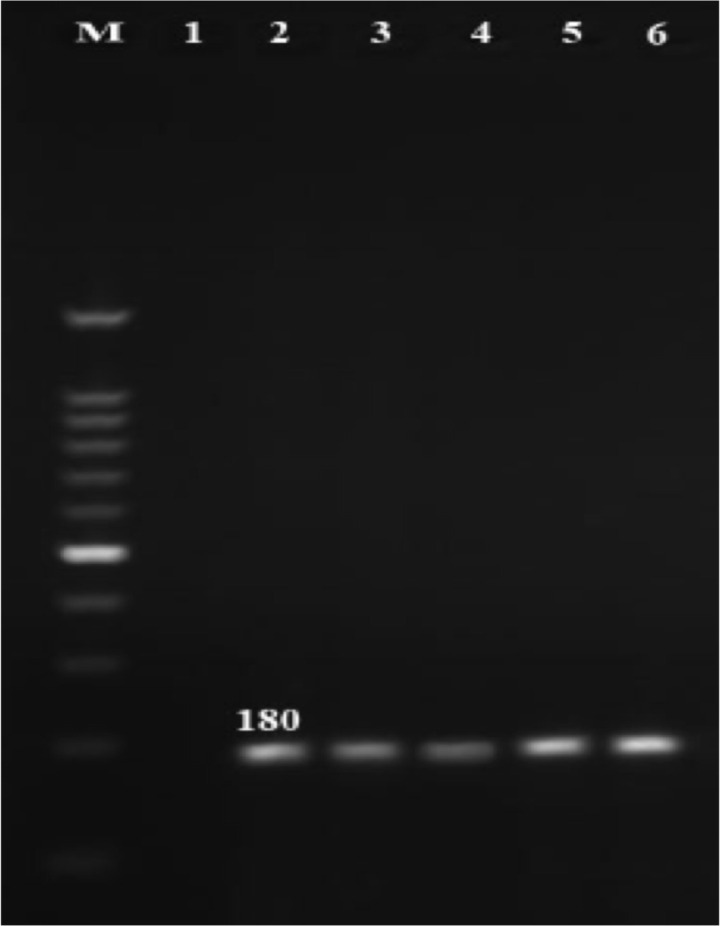
Polymerase chain reaction amplification of MOMP gene on agarose gel electrophoresis. M: DNA ladder (100 bp);1: negative control, 2: positive control;3–6: positive samples.

**Figure 2:**
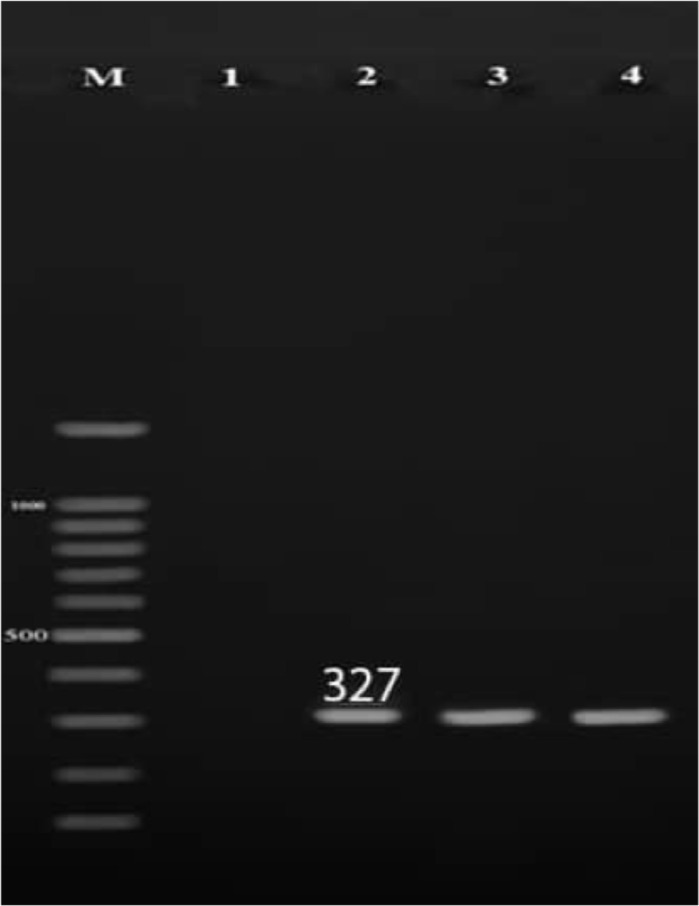
Polymerase chain reaction amplification of U.parvum(16SrRNA)on agarose gel electrophoresis. M: DNA ladder (100 bp);1: negative control, 2: positive control;3,4: positive samples.

**Prevalence of *C. trachomatis* and *M. genitalium:*** Out of the 50 semen specimens from infertile men, *C. trachomatis* and *U. parvum* were detected in 5(10%) and 6 (12%) of specimens by PCR, respectively (Fig. 1 and 2).

**Figure 3:**
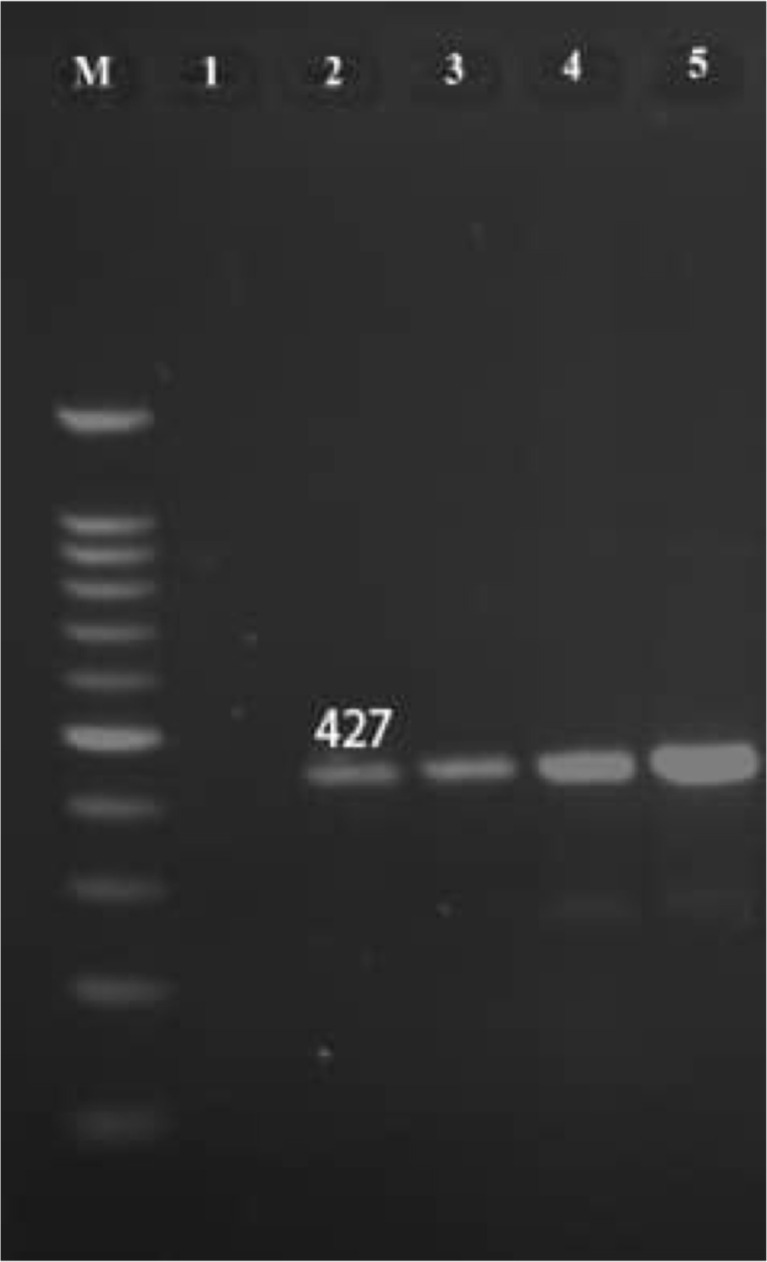
Polymerase chain reaction amplification of M.genitalium (16SrRNA) gene on agarose gel electrophoresis. M: DNA ladder (100 bp);1: negative control, 2: positive control;3–5: positive samples.

However, *M. genitalium* was not found in these cases. All of the semen specimens in the control group were negative for abovementioned microorganisms PCR. Also, out of 50 endocervical swabs from infertile women, 4 (8%) were positive for *M. genitalium*, and *C. trachomatis* was found in 7 (14%) of swab samples (Fig. 1 and 3).

However, *U. parvum* was not found in these specimens. In the control group, all of the endocervical swabs were negative for aforementioned microorganisms PCR. The distribution of the detected species of *C. trachomatis* and *M. genitalium* (Table 2).

**Table 2:** Frequency of *C. trachomatis, U. parvum* and *M. genitalium* by PCR in infertile and fertile men and women 9N=50)

Microorganism	Infertile men (n=50)	Healthy men (n=50)	Infertile women (n=50)	Healthy women (n=50)
	
	N (%)	N (%)	N (%)	N (%)
*C. trachomatis*	5(10%)	0%	7(14%)	0(%)
*U. parvum*	6(12%)	0%	0%	0%
*M. genitalium*	0%	0%	4(8%)	0%

Note: variables were tested by Chi-Square test

**The relationship between the detection of *C. trachomatis* and *U. parvum* and sperm variables**: Semen analysis was performed using the methods by the World Health Organization to determine: volume, motility, pH, sperm concentration. The mean values of sperm concentration, normal forms, and progressive motility were significantly lower in infertile men than in healthy men (P= 0.001) (Table 3).

**Table 3:** Comparison of seminal variables in infertile and fertile men

Variable	Infertile group (n=50)	Fertile group (n=50)	*P* value
	(Mean ± SD)	(Mean ± SD)	
PH	7.1 ± 0.8	7.0 ± 0.06	0.5
Volume (41)	3.1 ± 1.5	3.2 ± 1.4	0.5
Sperm concentration (×106/mL)	53± 30	65± 5	0.001
Total motility (%)	27.2 ± 17.3	53.1± 3.5	0.000
Normal forms (%)	5.9 ± 1.3	12.5 ± 1.7	0.001

Note: Values are means (±Standard Error), Unless indicated, variables were tested by Chi-Square test.

Also, to identify the effects of *C. trachomatis* and *U. parvum* on semen quality, semen variables were compared between the infected infertile men and uninfected infertile men. The motility showed a significant difference between the infected groups and uninfected groups (P= 0.001) (Table 4).

**Table 4:** Association (mean ± SD) of *C. trachomatis* and *U. parvum* infections on seminal variables in infertile men

Variable	*C. trachomatis(n=5)*	*U. parvum(n=6)*	Uninfected(n=39)
	mean ± SD(*P* value)	mean ± SD(*P* value)	mean ± SD(*P* value)
PH	7.0 ± 0.09 (0.5)	7.0 ± 0.09 (0.5)	7.1 ± 0.1
Volume(41)	3.0 ± 1.5 (0.6)	3.0 ± 1.5 (0.6)	3.1 ± 1.5
Sperm count(×10^6^ml)	50.0± 38 (0.7)	51± 38 (0.7)	55.4 ± 36.01
Progressive motility (%)	21.0± 10.02 (0.001)	21± 10.01 (0.001)	28.8± 13.6
Normal forms (%)	6.5 ± 1.2 (0.1)	6.5 ± 1.2 (0.1)	6.7 ± 1.8

Note: Values are means (Standard Error). #Unless indicated, variables were tested by Chi-Square test.

## Discussion

*C. trachomatis* and *M. genitalium* are caused by a widespread sexually transmitted infection such as infertility (12). Infections caused by these microorganisms are often asymptomatic. The exact mechanisms that genitourinary microorganisms affecting human fertility potential remain unknown (17). Chlamydial infections are a major burden on public health, especially in developed and developing countries that affect male and female fertility potential.

Mechanisms of the effect of this microorganism on male fertility may interact with sperm cells through lipopolysaccharide and consequently release reactive oxygen species that can induce apoptosis of sperm cells, obstructive azoospermia, directly damaging spermatogenesis and function of the mature spermatozoon, epithelial damage, producing inflammatory cytokines (4, 13, 18). In women, these infections can ascend beyond the cervix leading to PID and tubal pathology infections. The persistence of these infections also leads to long-term pathological immune responses and consequently tissue damages to the fallopian tube and female infertility (19). *M. genitalium* is a sexually transmitted organism that is often asymptomatic and associated with reproductive tract syndrome in women and men. This microorganism attached to the midpiece vesicle of the spermatozoon and carried by a sperm may be involved in sperm motility, which can lead to impaired sperm quality and male infertility (20, 21). Also, *M. genitalium* can bind to the epithelium of the vagina, ectocervix, endocervix, and fallopian tube. Persistence in these tissues stimulates inflammatory responses from the infected epithelium and consequently increases the risk of PID, tubal-factor infertility (22). Also, *Ureaplasma* spp. like *Mycoplasma* spp., are frequently isolated from the genitourinary tract and associated with genitourinary tract infections. This suggests that these infections have a potential role in infertility to prevent blastocyst from implantation by producing neuraminidase.

Besides, changing the pH of the vagina causes abortion, and disturbs the physiological properties of the vaginal fluid and sperm penetration decreases the count and efficiency of the sperm (23). Since the treatment of infertility has high financial costs, expansion of the screening programs for detecting these microorganisms is essential. The purpose of the present study was to investigate the prevalence of *C. trachomatis, U. parvum*, and *M. genitalium* in infertile couples and the effect of these infections on semen parameters. There is considerable variability in the rate of *C. trachomatis* infection among infertile males and females. In our study, the prevalence of *C. trachomatis* isolates of endocervical swabs and semen samples from the infertile group were 12%. Present results were relatively close to previous studies in Iran (15.3%) and Nigeria (9.6%) but were lower than a study in Tunisia (43.3%) (24–26). In the current study, *M. genitalium* was detected in (8%) of infertile women. However, *U. parvum* was not found in these cases. The frequency of *M. genitalium* isolates in our study was close to previous studies by Tomusiak. et al, and Mousavi et al., but another report showed that the prevalence of *M. genitalium* was lower than our study (24, 27, 28). In our study, *M. genitalium* was not found in infertile men, which is consistent with other studies (29). Also, our result showed that *U. parvum* was detected in 12% of infertile men that are similar to a study by Abusarah (30). This is higher than the prevalence reported in similar studies from Tunisia (4.2%) and Iran (3%), and lower than reported from China (19.2%) (31, 32). Differences in reports may be due to differences in laboratory methods, geographical and cultural characteristics of the countries, variation in the population studied (33). We also compared the prevalence of these infections in healthy and infertile groups. Some studies have shown that the prevalence of *C. trachomatis* and genital *Mycoplasma* spp. are higher in the infertile group than in the healthy group (16, 32, 34, 35).

Our study showed that the rate of detection of these microorganisms in samples of the infertile group was higher than the healthy group (Table 2), which is similar to previous studies (16, 31, 32, 34, 35). However, other studies have reported there was no difference in the prevalence of these infections in the infertile and healthy groups (36, 37).

A conclusion of all of these studies, due to the diversity of the population and the geographical and cultural characteristics of the countries and multiple sexual partners is difficult (33). In addition to the prevalence of these microorganisms, we evaluated the relationship between these bacteria and sperm quality. Previous studies on the effects of these infections on semen quality showed conflicting results. Some studies have reported a high incidence of Chlamydial and genital *Mycoplasma* infections among infertile males, and the negative effect of these infections on semen parameters (5, 18, 27). But other reports showed that there is no relationship between these microorganisms and semen quality (37). The present study showed that *C. trachomatis* and *U. parvum* are associated with impaired sperm motility and this is consistent with some of previous studies (5, 18, 27). This difference in reports can be explained by the capability of these microorganisms to attach to sperm and influence on vitality, motility, morphology, cellular integrity, or host factors and cellular interactions (23, 33).

This study showed that the *C. trachomatis* and *M. genitalium* seem to be widespread among infertile couples in Khuzestan Province (south-west of Iran). Also, our results demonstrated that the *C. trachomatis* and *U. parvum* may have a negative effect on semen quality, which can lead to infertility. Therefore, clinicians should consider these infections in the treatment of infertile couples.

As a limitation, we did not determine the bacterial loads of the infected specimens, by quantitative methods such as real-time PCR, and also the number of our population was low, the larger sample size is required for a firm conclusion on the effect of these bacteria on infertility. It is needed to confirm the results of PCR and electrophoresis by sequencing which unfortunately was not done because of not considering as initial goals of the study in the financial resources of the plan. It is an important limitation in the interpretation of the results.
